# High-resolution quantitative 3D T2 mapping allows quantification of changes in edema after myocardial infarction

**DOI:** 10.1186/1532-429X-15-S1-P181

**Published:** 2013-01-30

**Authors:** Haiyan Ding, Michael Schär, MM Zviman, Henry R Halperin, Roy Beinart, Daniel A Herzka

**Affiliations:** 1 Biomedical Engineering, Tsinghua University, Beijing, China; 2 Biomedical Engineering, Johns Hopkins University School of Medicine, Baltimore, MD, USA; 3 Medicine, Cardiology, Johns Hopkins University School of Medicine, Baltimore, MD, USA; 4 Russell H. Morgan Department of Radiology and Radiological Science, Johns Hopkins University School of Medicine, Baltimore, MD, USA; 5 Philips Healthcare, Cleveland, OH, USA

## Background

T2 values are related to the tissue water content, providing useful diagnostic information in cardiac diseases, especially in acute states such as myocardial infarction (MI). Visualization of edema, its regional distribution, and extent in acute and chronic heart disease may be useful as a diagnostic tool and help in guiding treatment in the patients with heart disease. Recently, edema detection (T2 elevation) using quantitative T2 maps has been shown more robust than qualitative clinical T2W imaging.

Hypothesis: Whole heart coverage T2 mapping makes the global edema distribution available for quantitative myocardial edema assessment in the context of subacute MI.

## Methods

Under IACUC-approved protocol, MI was induced in swine (N = 4) by 120 min occlusion of the mid LAD. Imaging was carried out 2 to 9 days post MI using an Achieva 3T TX system (Philips Healthcare, Best, Netherlands). Normal animals (N=4) were imaged for reference T2 values. T2-mapping of the ventricles was carried using previously presented free-breathing 3D sequence. Post-contrast 3D Late Gadolinium enhancement with phase sensitive inversion recovery (PSIR) images were acquired ~30min post infusion of 0.2 mmol/kg of Magnevist. Both sequences were acquired in high-resolution (~1.0x1.25x3 mm3 for PSIR, 1.25x1.25x5 mm3 for T2 Maps) during free-breathing using independent respiratory navigator gating.

3D T2 maps were calculated per voxel using linear regression of the log of the signal. Pixels with poor fits (corr. coeff. R2<0.9) were rejected. The left ventricle (LV) was manually segmented, excluding papillary muscle and epicardial/endocardial boundaries.

## Results

**Figure 1:**
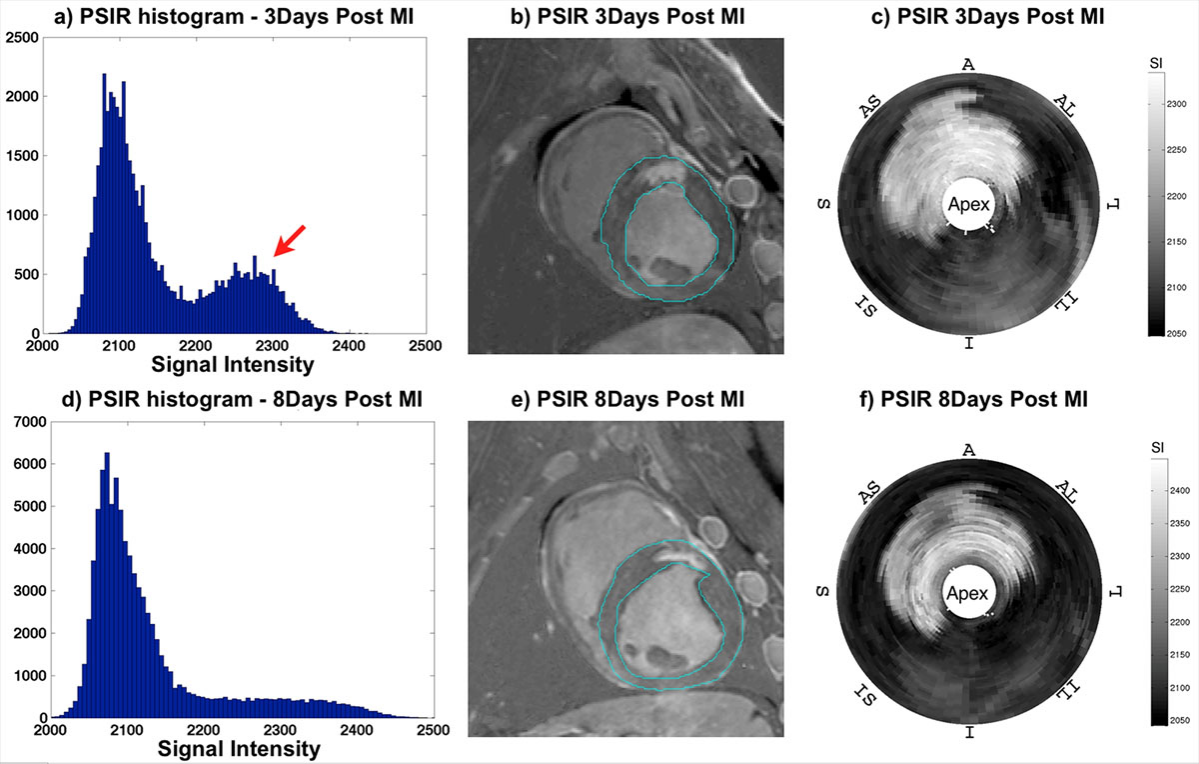
Representative PSIR images along with whole-heart histograms and Bull's Eye plots from 3/8 Days post MI

**Figure 2:**
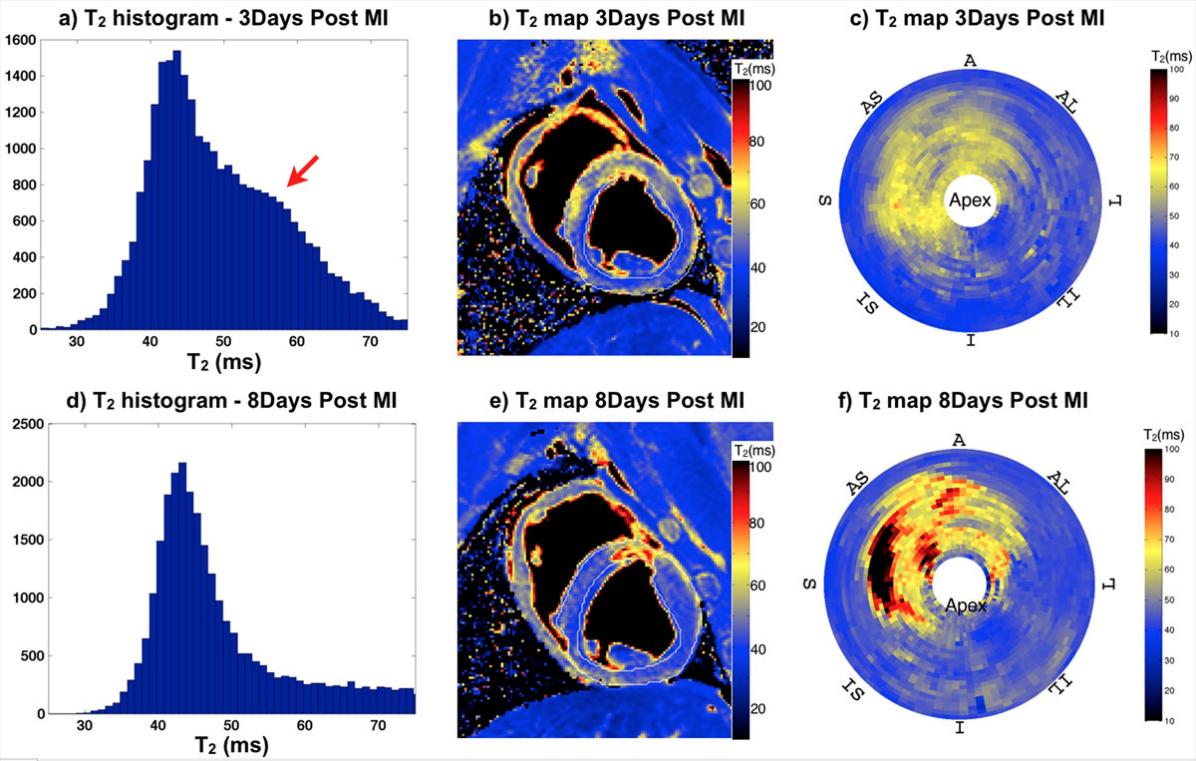
Matching representative T2 Maps, whole-heart histograms and Bull's Eye plots for the same animal in Figure 1.

MI with edema was observed in all animals; 3 animals displayed evidence of hemorrhage/clotting injuries as evidenced by microvascular obstruction and concomitantly reduced T2. Figs. 1 and 2, show representative short-axis post MI PSIR and T2 map images, respectively, along with whole LV volume histograms and Bull's Eye plots. Data demonstrate the dissipation of edema (increased T2) over time (red arrows). PSIR viability volumes display the extent of infarct, with infarct size decreasing from 3 days post MI to 8 days. T2 and edema increase in the same time span.

## Conclusions

The high resolution quantitative T2 maps enables monitoring of physiological changes over time, providing an objective assessment on the evolution of edema post MI.

## Funding

This work was funded in part by AHA-11SDG5280025.

